# Effects of Mobility-Fit, a tailored multicomponent physical activity program with upper-limb emphasis, on strength, mobility and fall risk among older adults in long-term care: a cluster randomised controlled trial

**DOI:** 10.1093/ageing/afaf349

**Published:** 2025-12-15

**Authors:** Ziwei Zeng, Jing Wen Pan, Kimberley S van Schooten, Kin-Tung Chan, Chi-Tat Fung, Yijian Yang

**Affiliations:** Department of Sports Science and Physical Education, The Chinese University of Hong Kong, N.T., Hong Kong, China; Department of Sports Science and Physical Education, The Chinese University of Hong Kong, N.T., Hong Kong, China; Neuroscience Research Australia, Randwick, New South Wales, Australia; School of Population Health, University of New South Wales, Sydney, New South Wales, Australia; Hong Kong Sheng Kung Hui Welfare Council Limited, Kowloon, Hong Kong, China; Hong Kong Sheng Kung Hui Welfare Council Limited, Kowloon, Hong Kong, China; Department of Sports Science and Physical Education, The Chinese University of Hong Kong, N.T., Hong Kong, China; Jockey Club Institute of Ageing, The Chinese University of Hong Kong, N.T., Hong Kong, China

**Keywords:** care home, exercise, strength, quality of life, safe mobility, older people

## Abstract

**Background:**

Older adults in long-term care (LTC) face accelerated functional decline due to frailty, affecting mobility and quality of life (QoL). Upper-body strength is crucial for daily activities and injury prevention, yet conventional physical activity (PA) programs often overlook it. This cluster randomised controlled trial evaluated Mobility-Fit, a 12-week multicomponent PA program emphasising upper-limb and core strength among LTC residents.

**Methods:**

Twenty LTC facilities were randomised into the Mobility-Fit (10 facilities, *n* = 73) or control (10 facilities, *n* = 73) group. Mobility-Fit included agility, balance and resistance exercises (3 times/week, 45 minutes), tailored to participants’ functional capacity. The control group received standard care with lower-limb exercises, delivered with the same frequency. Outcomes included upper- and lower-limb strength, postural sway, reaction time, mobility (Short Physical Performance Battery), frailty (FRAIL-Nursing Home), fall risk (Longitudinal Aging Study Amsterdam fall risk profile questionnaire, LASA) and QoL [EuroQol (EQ)-5D]. An intention-to-treat analysis with generalised estimating equations was conducted.

**Results:**

Participants (median age = 86, 60% female) exhibited high adherence (83.3%). Mobility-Fit demonstrated superior improvements in elbow extension strength [β = 0.50, 95% confidence interval 0.26–3.89] and QoL (EuroQol five-dimension questionnaire utility: *β* = 0.17, *P* < .001; EQ-Visual Analogue Scale: *β* = 7.13, *P* = .006). Both groups improved lower-limb strength (knee extension: *β* = 2.72, *P* < .001) and mobility (Sit-To-Stand time: *β* = −3.69, *P* < .001), with a reduced LASA score (*β* = −1.20, *P* < .001).

**Conclusions:**

Mobility-Fit effectively enhanced upper-limb strength and QoL in LTC residents. Both groups showed improvements in lower-limb strength and mobility, highlighting the universal benefits of structured PA. The program addresses a critical gap by demonstrating the value of integrating upper-body training into standard care to promote holistic well-being.

## Key Points

- Mobility-Fit, a tailored multicomponent physical activity program, significantly improved upper-limb strength and quality of life in long-term care residents compared to standard lower-limb training.
- The Mobility-Fit program demonstrated high adherence (92%) and safety (no adverse events), supporting its feasibility for real-world implementation in long-term care settings.
- Subgroup analyses highlighted differential benefits: non-frail participants improved both physical function and quality of life, while frail individuals primarily enhanced quality of life, emphasising the need for frailty-specific interventions.

## Introduction

The global population is ageing rapidly, with significant increases in both the number and lifespan of older adults [1]. In Hong Kong, individuals aged 65 and older are projected to comprise 29% of the population by 2036 [2]. Ageing is accompanied by declines in physical and mental health, leading to frailty [3], a syndrome characterised by reduced muscle mass, strength and functional reserve [4], which increases the risk of adverse outcomes such as falls and hospitalisation [3, 4].

Older adults in long-term care (LTC) facilities face distinct challenges, exhibiting higher rates of multimorbidity, cognitive deficits and depression than community-dwelling counterparts [5, 6]. They also spend over 80% of their waking hours sedentary [7], which may accelerate sarcopenia and functional decline [6, 8]. Upper-body strength is essential for performing activities of daily living (ADLs) [9, 10] and mitigating fall-related injuries through protective responses [11, 12]. Despite this importance, most LTC exercise interventions focus predominantly on lower-limb strength and balance [13].

Multicomponent physical activity (PA) programs that combine strength, aerobic, balance and flexibility training have shown promise in improving physical function in older adults [13]. However, upper-body strengthening is often neglected, despite its potential to enhance mobility and protective responses during the loss of balance [9, 10, 13]. Our prior research revealed that over 20% of falls in LTC resulted from inadequate postural control during transfers (e.g. getting up or sitting down), and one-third involved head impact despite active attempts to arrest falls using upper limbs [11, 12]. This suggests that PA programs should target both lower-limb, upper-limb and core functions to promote safe transferring and reduce fall-related injuries.

To address these gaps, we developed Mobility-Fit, a 12-week multicomponent PA program for LTC residents. Mobility-Fit emphasises ADL performance and fall prevention by integrating upper-limb and core exercises with lower-limb training, tailored to individual abilities [14, 15]. Although a pilot study demonstrated feasibility and potential benefits [15], robust evidence is needed. Therefore, this cluster randomised controlled trial (RCT) aimed to evaluate the effects of Mobility-Fit, compared to a standard lower-limb strengthening program, on upper- and lower-limb strength, trunk stability, mobility, fall risk, frailty and quality of life (QoL) in LTC residents. The secondary aim was to examine frailty-specific effects, which could inform personalised approaches to training. We hypothesised that (i) Mobility-Fit would improve upper-limb strength, mobility and QoL more effectively than standard lower-limb training and (ii) its effects would differ by frailty status, with non-frail participants showing greater functional benefits.

## Methods and analysis

### Study design

This two-arm, parallel-group, cluster RCT compared the Mobility-Fit program to a standard lower-limb strengthening program among older adults in LTC facilities. The study adhered to the Declaration of Helsinki and Good Clinical Practice guidelines. The detailed study protocol and main outcomes are described elsewhere [14]. This protocol adheres to the SPIRIT guidelines and is registered with the Chinese Clinical Trial Registry (ChiCTR2300072709). The report followed the CONSORT statement. Ethical approval was obtained from the Institutional Research Ethics Committee (Ref. No.: SBRE-21-0413 and CREC-2022-459), and informed consent was obtained from all participants.

### Participants and recruitment

Participants were recruited from LTC facilities. Recruitment occurred at care facilities operated by the Hong Kong Sheng Kung Hui (HKSKH) Multi-disciplinary Outreaching Support Team for the Elderly (MOSTE). Physiotherapists (PTs) identified eligible participants based on their physical capacity and willingness to participate in the study.

The sample size was determined through a priori power analysis using G^*^Power 3.1.9.2. For the primary objective, a repeated-measures ANOVA with 90% power, an alpha of 0.05 and a moderate effect size (f = 0.3) required 80 participants (40 per group). To account for clustering effects [16], we applied a design effect formula: 1 + (*n* − 1)^*^ρ, where *n* (cluster size) = 8 and *ρ* (intra-cluster correlation coefficient) = 0.05 [17]. This yielded a design effect of 1.35, inflating the sample size to 108 participants across 14 clusters. After accounting for a 25% dropout rate, the sample size was increased to 144. To accommodate subgroup analyses, the final minimal sample size was set at 160 participants from 20 LTC facilities (clusters) [14].

Inclusion criteria were: (i) aged 65 years or older and residing in an LTC facility, (ii) able to rise from a chair and stand unaided for at least 20 seconds and (iii) having medical clearance for PA. Exclusion criteria included: (i) severe cognitive impairment (inability to follow simple instructions), (ii) legal blindness or (iii) bedridden status.

### Randomisation and blinding

Twenty LTC facilities were stratified by facility size and care level to ensure balanced allocation across the groups. Facilities were randomised 1:1 to the Mobility-Fit (intervention) or standard care (control) group using a computer-generated algorithm by an independent statistician. Outcome assessors were blinded to group allocation, although blinding of intervention instructors was not feasible. Participants were not informed of their group assignment.

### Intervention

#### Intervention group

The Mobility-Fit program was delivered three times per week for 12 weeks, with each session lasting approximately 45 minutes and comprising the following components [14]:

- Warm-up (5 minutes): joint mobility exercises (e.g. shoulder circles, seated marching).
- Main exercise phase (40 minutes):

Agility training: ball toss, random foot placement, and multitasking activities.
Postural coordination and balance training: Frequent lumbar extension and shoulder external rotation; static and dynamic balance training.
Resistance training: upper-limb: triceps extensions and wrist curls; Core: exercises to promote trunk stability in various positions; Lower-limb: hip abduction and extension, heel raises, and hamstring exercises.

- Cool-down (2–5 minutes): stretching and breathing exercises.

Exercises were tailored to participants’ mobility, strength, stamina and cognitive function, with progression as appropriate (Supplementary Appendix S1). Participants with limited lower-limb strength performed seated exercises with resistance bands.

#### Control group

The control group received a lower-limb strengthening program, which is part of the standard care provided by the MOSTE. This program followed a standardised protocol used by the PTs across their managed LTC facilities, focusing on seated quadriceps extension, ankle dorsiflexion and standing hip abduction exercises, without upper-limb and core training. This design allowed us to isolate the specific effect of adding these upper-body and multicomponent elements. These sessions were similarly held three times per week for 45 minutes. Both interventions were delivered by trained PTs and recreation workers.

### Data collection and outcome measures

Data were collected at each LTC facility. Demographic information (age and sex) and body mass index (BMI) were obtained from the facility health records. Two well-trained research assistants administered outcome measures at baseline and after the 12-week intervention, with an additional assistant present for safety.

Adherence was calculated as the percentage of sessions attended out of the 36 sessions offered, based on attendance records kept by the facility staff. Adverse events were systematically monitored by the intervention instructors during each session. Any undesirable symptom, complaint or incident experienced by a participant was recorded by PTs and reported to the principal investigator.

#### Primary outcomes

The primary outcomes were selected to capture the multi-dimensional nature of physical capacity related to safe mobility. Although a family of primary outcomes was pre-specified, upper-limb strength (elbow flexion and extension) was designated as the key primary outcome to directly test the study’s central hypothesis regarding the benefit of upper-body training. The remaining measures provided an assessment of overall physical function.

- Upper-limb strength: (a) Elbow flexion and extension strength: evaluated using the Hoggan microFET2 hand-held dynamometry (HHD) (Hoggan Scientific, Salt Lake City UT, USA) [18], which has a measurement range of 0–300 lb and demonstrates high reliability and validity [19]. Participants were seated with their dominant elbow flexed at 90° and resting on a table. The HHD was placed against the inside and outside of the distal forearm. They were instructed to press maximally against it, holding the contraction for 3 seconds. The highest value from two trials was recorded. (b) Handgrip strength: measured using a HHD (5001 Grip-A; Takei, Japan) [20]. While standing, participants let their dominant arm hang naturally and squeezed the dynamometer as forcefully as possible for 3 seconds. The highest value from two trials was recorded. This test demonstrates good to excellent reliability [21, 22].
- Lower-limb strength: Quadriceps strength was assessed using the Hoggan microFET2 HHD [23]. Participants sat on an adjusted chair so that their feet did not touch the ground. The HHD was positioned on the anterior aspect of the dominant leg’s distal tibia, and participants performed a maximal knee extension for 3 seconds. The highest value from three trials was recorded.
- Reaction time: Assessed using the Physiological Profile Assessment, a valid and reliable tool for evaluating fall risk in older adults [24]. After five practice trials, participants performed 10 formal trials. The average reaction time was used for data analysis.
- Postural sway: Participants stood with eyes open for 30 seconds while a sway metre, secured at waist level, recorded total sway in the antero-posterior and medio-lateral directions [24]. The mean value from two trials was used.
- Mobility function: Evaluated using the Short Physical Performance Battery (SPPB), a well-established and valid measure of functional status [25, 26]. The SPPB includes a 4-m walk test, a five-time Sit-To-Stand (STS) test, and balance tests in side-by-side, semi tandem and tandem standing positions. Total scores range from 0 to 12, with higher scores indicating better mobility.
- Fall risk: Assessed using the Longitudinal Aging Study Amsterdam (LASA) fall risk profile questionnaire, a validated tool for predicting falls [27, 28]. This nine-item questionnaire yields a total score ranging from 0 to 30, where higher scores indicate higher risk.

#### Secondary outcomes

- QoL: Evaluated using the Hong Kong version of the EuroQol five-dimension questionnaire (EQ-5D)-5L instrument and the EuroQol Visual Analogue Scale (EQ-VAS), which is a reliable and valid generic instrument for describing health status across various populations and settings [29, 30]. Utility scores were derived using Hong Kong-specific weights [31], and the EQ-VAS recorded self-rated overall health on a vertical scale from 0 (worst) to 100 (best) [29].
- Frailty: Measured using the 7-item FRAIL-Nursing Home (NH) scale, which shows good agreement with more complex frailty instruments [32]. The scale assesses fatigue, resistance, mobility, incontinence/disease, weight loss, eating style and assistance with dressing. Items are scored 0, 1 or 2, yielding a total score from 0 to 14. Participants were classified as non-frail (score 0–1) or frail (score ≥2) [33].

### Data analyses

Analyses were completed using R software (version 4.4.3) and the *geepack* package [34]. Statistical significance was set at *P* < .05. Analyses followed the intention-to-treat (ITT) principle, including all participants based on their original group assignment. A per-protocol analysis, which included only participants who attended the post-intervention assessment, was also conducted as a sensitivity analysis. Descriptive statistics characterised baseline participant demographics and outcome measures. Missing data were addressed using multiple imputation by chained equations with 20 imputations, and results were pooled using the *mice* package in R [35].

To account for the clustered nature of the data and repeated measurements, generalised estimating equations (GEEs) with an exchangeable correlation structure were employed, specifying LTC facilities as clusters. The models were adjusted for age, sex, BMI, mobility aid use and baseline values of the respective outcome variable. For each outcome, the GEE model examined the Group × Time interaction effect and the main effects of Group and Time. Where applicable, Bonferroni correction was applied to adjust for multiple comparisons. Subgroup analyses were performed based on frailty status (Supplementary Appendix S2). Effect sizes were estimated using Cohen’s d, interpreted as small (0.2), medium (0.5) and large (0.8) [36].

## Results

### Baseline characteristics

**Figure 1:**
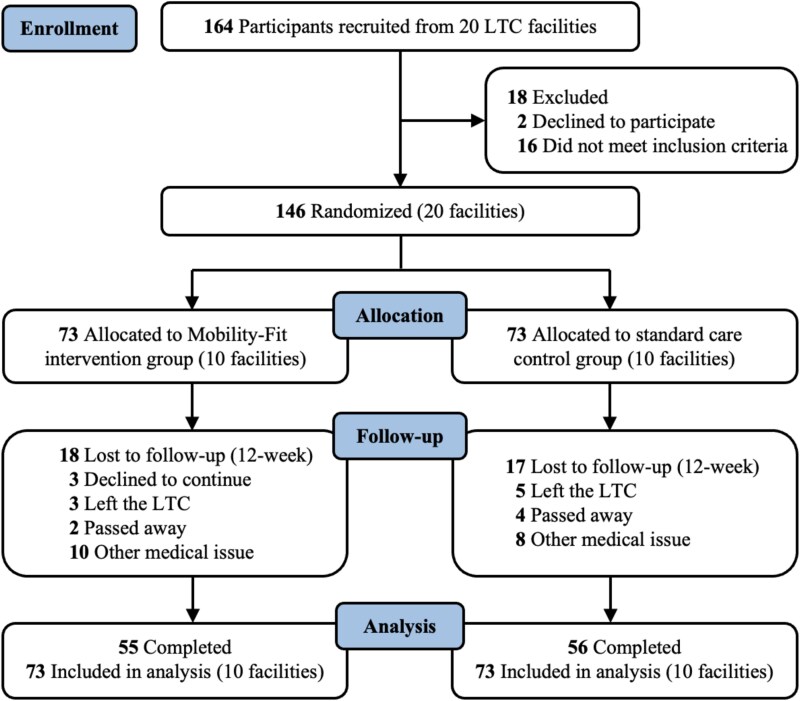
CONSORT flow diagram.

Of 164 enrolled participants from 20 LTC facilities, 146 met eligibility criteria and were randomly assigned to the Mobility-Fit intervention (*n* = 73) or the standard care control group (*n* = 73) (Figure 1).

The overall median age was 86 years, with 60% female participants and a median BMI of 21.6 kg/m^2^. Additionally, 62% of participants used walking aids (Table 1).

**Table 1:** Baseline characteristics of participants.^a^

	Total (*n* = 146)	Control (*n* = 73)	Intervention (*n* = 73)
Age (years)	86 (12)	86 (11)	84 (12)
Sex			
Female	87 (59.6%)	47 (64.4%)	40 (54.8%)
Male	59 (40.4%)	26 (35.6%)	33 (45.2%)
Body height (cm)	156.1 ± 9.7	154.5 ± 9.3	157.6 ± 9.9
Body weight (kg)	53.6 ± 10.5	53.6 ± 10.9	53.5 ± 10.2
BMI (kg/m^2^)	21.6 (4.8)	22.1 (5.0)	21.0 (4.5)
Walking aid used			
Independent	55 (37.7%)	26 (35.6%)	29 (39.7%)
Cane	67 (45.9%)	33 (45.2%)	34 (46.6%)
Walker	24 (16.4%)	14 (19.2%)	10 (13.7%)
Primary outcomes			
Elbow flexion strength (kg)	6.7 (4.3)	6.3 (4.5)	7.2 (4.3)
Elbow extension strength (kg)	5.4 (2.8)	5.8 ± 2.3	5.6 ± 2.2
Knee extension strength (kg)	11.4 ± 4.4	11.1 ± 4.3	11.7 ± 4.5
Handgrip-R (kg)	12.8 (7.5)	13.4 ± 4.6	14.4 ± 6.1
Handgrip-L (kg)	12.1 (7.0)	11.6 (6.0)	13.2 (7.4)
Reaction time (s)	0.458 (0.360)	0.478 (0.347)	0.441 (0.378)
Postural sway-AP (mm)	22.25 (11.75)	22.50 (9.50)	21.50 (14.00)
Postural sway-ML (mm)	31.75 (21.38)	32.00 (20.50)	30.50 (22.50)
STS time (s)	20.7 (11.1)	20.4 (10.7)	21.1 (12.1)
Walking speed (m/s)	0.42 (0.30)	0.39 (0.32)	0.43 (0.31)
SPPB-Total (point)	5 (3)	5 (2)	6 (3)
SPPB-STS (point)	1 (1)	1 (1)	1 (1)
SPPB-Gait (point)	1 (0)	1 (0)	1(1)
SPPB-Balance (point)	2.5 (2)	2 (2)	3 (2)
LASA (point)	5 (5)	4 (6)	5 (4)
Secondary outcomes			
EQ-5D utility score	0.81 (0.36)	0.84 (0.25)	0.74 (0.46)
EQ-VAS	70 (35)	70 (35)	70 (30)
FRAIL-NH (point)	1 (2)	1 (2)	1 (2)
Attendance session	30 (16)	25 (20)	33 (11)

^a^Continuous variables with normally distributed were presented in mean ± SD; continuous variables with non-normally distributed were presented in median (interquartile range); categorical variables were presented in frequency (percentage). AP, antero-posterior; FRAIL-NH, 7-item FRAIL-Nursing Home Scale; L, left; ML, medio-lateral; R, right.

### Effects of the intervention

#### Primary outcomes

**Figure 2:**
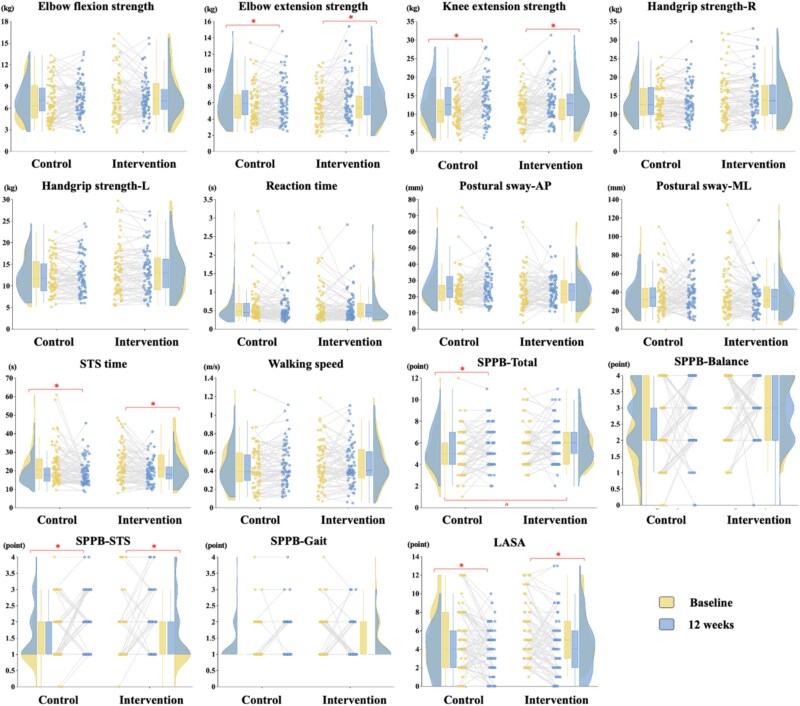
Primary outcomes by intervention group over time. The density plot reflects a kernel density estimate, illustrating the distribution of data across different values—wider sections indicate higher data concentration. The thick line within each box plot represents the median, while the box boundaries show the interquartile range. Paired scatter plots illustrate individual changes over time. ^*^ denotes significant within-group differences based on pairwise comparisons; ^ denotes significant between-group differences at baseline based on pairwise comparisons. AP, antero-posterior; L, left; ML, medio-lateral; R, right.

The GEE model identified a statistically significant Group × Time interaction for elbow extension strength {*β* = 0.50 [95% confidence interval (CI) 0.26, 3.89], *P* = .049, adjusted} (Table 2). Pairwise comparisons revealed significant increases in elbow extension strength from baseline to 12 weeks in both groups [intervention: *β* = 1.00 (95% CI 0.70, 1.30), adjusted *P* < .001; control: *β* = 0.50 (95% CI 0.10, 0.89), adjusted *P* = .014], with greater improvements in the intervention group (Figure 2). No significant interactions were found for other primary outcomes.

**Table 2:** Effects of the mobility-fit training on primary and secondary outcomes by unadjusted and adjusted generalised estimating equation model.^a^

Variable	Unadjusted generalised estimating equation model							Adjusted generalised estimating equation model						
	Group × Time interaction		Group (Intervention vs. Control)		Time (12 weeks vs. Baseline)		Cohen’s d	Group × Time interaction		Group (Intervention vs. Control)		Time (12 weeks vs. Baseline)		Cohen’s d
	*β* (SE)	*P*	*β* (SE)	*P*	*β* (SE)	*P*		*β* (SE)	*P*	*β* (SE)	*P*	*β* (SE)	*P*	
Elbow flexion strength (kg)	−0.50 (0.65)	.446	0.71 (0.50)	.153	0.29 (0.44)	.516	0.08	−0.54 (0.33)	.100	0.17 (0.14)	.202	0.34 (0.26)	.193	0.24
Elbow extension strength (kg)	0.50 (0.60)	.402	−0.18 (0.39)	.648	0.49 (0.42)	.245	0.14	0.50 (0.26)	**.049**	−0.11 (0.11)	.291	0.50 (0.20)	**.014**	0.31
Knee extension strength (kg)	−0.76 (1.36)	.577	0.48 (0.80)	.543	2.60 (0.92)	**.005**	0.06	−0.79 (0.83)	.342	−0.05 (0.28)	.849	2.72 (0.71)	**<.001**	0.26
Handgrip strength-R (kg)	−0.10 (1.30)	.941	0.94 (0.95)	.323	0.24 (0.81)	.769	0.16	−0.07 (0.51)	.886	0.04 (0.11)	.729	0.24 (0.36)	.501	0.02
Handgrip strength-L (kg)	0.08 (1.03)	.941	1.10 (0.83)	.184	−0.04 (0.54)	.940	0.24	0.14 (0.47)	.783	0.16 (0.12)	.191	−0.07 (0.31)	.829	0.16
Reaction time (s)	0.02 (0.12)	.875	0.02 (0.10)	.879	−0.07 (0.10)	.456	0.08	0.02 (0.06)	.730	−0.01 (0.03)	.960	−0.07 (0.05)	.121	0.08
Postural sway-AP (mm)	−2.51 (2.57)	.330	−0.83 (2.03)	.683	2.41 (1.60)	.132	0.32	−2.45 (1.54)	.112	−0.29 (0.55)	.596	2.43 (0.86)	**.005**	0.43
Postural sway-ML (mm)	−4.67 (5.86)	.425	2.31 (4.69)	.623	0.03 (2.54)	.992	0.12	−4.62 (3.29)	.160	0.97 (1.57)	.536	−0.04 (2.43)	.987	0.27
STS time (s)	−0.46 (1.97)	.815	0.62 (1.48)	.677	−3.58 (1.39)	**.010**	0.02	−0.37 (1.21)	.761	0.34 (0.49)	.491	−3.69 (0.73)	**<.001**	0.01
Walking speed (m/s)	−0.01 (0.06)	.859	0.03 (0.04)	.457	0.01 (0.04)	.887	0.09	−0.01 (0.03)	.655	−0.01 (0.01)	.925	0.01 (0.01)	.474	0.12
SPPB-Total (point)	−0.41 (0.47)	.390	0.63 (0.25)	**.012**	0.59 (0.28)	**.036**	0.12	−0.38 (0.30)	.203	0.16 (0.07)	**.036**	0.57 (0.20)	**.004**	0.21
SPPB-STS (point)	−0.04 (0.20)	.835	0.04 (0.12)	.722	0.26 (0.12)	**.035**	< 0.01	−0.05 (0.12)	.684	0.01 (0.02)	.684	0.27 (0.07)	**<.001**	0.07
SPPB-Gait (point)	−0.14 (0.15)	.345	0.15 (0.10)	.123	0.07 (0.11)	.531	0.02	−0.14 (0.08)	.077	0.02 (0.03)	.427	0.08 (0.05)	.148	0.34
SPPB-Balance (point)	−0.21 (0.26)	.429	0.46 (0.16)	**.006**	0.22 (0.17)	.190	0.24	−0.22 (0.18)	.229	0.17 (0.08)	**.024**	0.24 (0.14)	.091	0.07
LASA (point)	0.07 (0.82)	.934	−0.14 (0.07)	.053	−0.02 (0.03)	.427	0.14	0.10 (0.48)	.835	0.11 (0.18)	.521	−1.20 (0.30)	**<.001**	0.11
EQ-5D utility score	0.18 (0.08)	**.028**	−0.14 (0.07)	.054	0.05 (0.03)	.050	0.14	0.17 (0.04)	**<.001**	−0.05 (0.03)	**.043**	−0.02 (0.02)	.327	0.71
EQ-VAS	7.24 (4.19)	.084	−0.85 (3.45)	.805	−2.71 (3.11)	.384	0.34	7.13 (2.58)	**.006**	−0.56 (1.30)	.665	−2.61 (1.74)	.133	0.47
FRAIL-NH (point)	0.24 (0.78)	.760	0.06 (0.62)	.927	0.09 (0.45)	.837	0.13	0.24 (0.46)	.608	−0.04 (0.11)	.683	0.10 (0.20)	.610	0.14

^a^Adjusted by age, sex, BMI, walking aid used and baseline values. Bold *P*-value represents statistical significance. AP, antero-posterior; FRAIL-NH, 7-item FRAIL-Nursing Home Scale; L, left; ML, medio-lateral; R, right; SE, standard error.

The per-protocol analysis showed similar results (intervention group: *n* = 55; control group: *n* = 56), except for the interaction for elbow extension strength, which was no longer statistically significant [*β* = 0.60 (95% CI −0.04, 1.23), *P* = .064, adjusted] (Supplementary Appendix S3). However, the intervention group still showed significant within-group improvements in elbow extension strength at 12-week (*P* < .001), while the control group did not (*P* = .114).

#### Secondary outcomes

**Figure 3:**
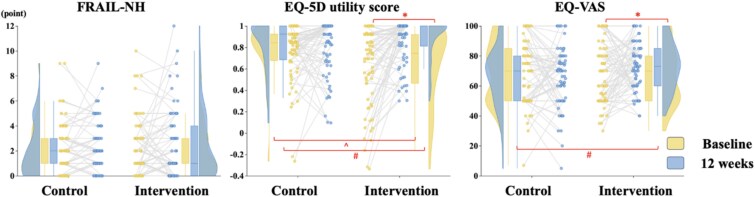
Secondary outcomes by intervention group over time. The density plot reflects a kernel density estimate, illustrating the distribution of data across different values—wider sections indicate higher data concentration. The thick line within each box plot represents the median, while the box boundaries show the interquartile range. Paired scatter plots illustrate individual changes over time. ^*^ denotes significant within-group differences based on pairwise comparisons; ^ denotes significant between-group differences at baseline based on pairwise comparisons; # denotes significant between-group differences at 12 weeks based on pairwise comparisons. FRAIL-NH, 7-item FRAIL-Nursing Home Scale.

The GEE model demonstrated significant Group × Time interactions for the EQ-5D utility score [*β* = 0.17 (95% CI 0.09, 0.25), *P* < .001, adjusted] and EQ-VAS [*β* = 7.13 (95% CI 2.07, 12.19), *P* = .006, adjusted] (Table 2). Pairwise comparisons indicated that the intervention group experienced significantly higher increases in both the EQ-5D utility score [*β* = 0.15 (95% CI 0.08, 0.22), adjusted *P* < .001] and EQ-VAS [*β* = 4.52 (95% CI 0.75, 8.29), adjusted *P* = .019] from baseline to 12 weeks (Figure 3). No significant interaction effects were observed for the FRAIL-NH score.

Subgroup analyses and their results are detailed in Supplementary Appendix S2.

### Attrition and adherence

The attrition rate was 23%, primarily due to hospitalisation or withdrawal. Adherence to the intervention was relatively high (*P* = .022) (Table 1). No adverse events were reported by the participants during the intervention.

## Discussion

Our results extend prior evidence supporting the efficacy of multicomponent PA programs in mitigating functional decline in older adults in LTC settings [13]. This trial provides three critical insights: (i) Mobility-Fit significantly enhanced elbow extension strength, addressing a gap in conventional LTC interventions by targeting upper-body function, which is essential for activities such as rising from a chair and performing protective arm responses during falls; (ii) both groups improved in lower-limb strength and mobility, underscoring the universal value of structured PA in frail populations and (iii) Mobility-Fit uniquely improved health-related QoL, regardless of frailty status, suggesting psychosocial benefits beyond physical gains.

The superior improvement in elbow extension strength observed in the Mobility-Fit group supports targeting both upper-body and core training, which are crucial for protective responses during falls [9–13], and can potentially reduce injury risk even when a fall is unavoidable. The non-significant interaction in the per-protocol analysis suggests that the effects may be adherence-related. Although both groups exhibited comparable improvements in lower-limb strength and STS performance, these gains may reflect the common emphasis on seated resistance training. These exercises have been shown to yield functional benefits even at low intensities in frail cohorts [37].

The lack of significant between-group differences for other primary outcomes may be explained by several factors. The use of an active control group that also engaged in supervised lower-limb exercise set a high threshold for demonstrating additional benefits. Furthermore, the 12-week duration and tailored intensity of the intervention, while ensuring feasibility and safety, may have been insufficient to overcome the profound physical deficits and limitations in this advanced-age, high-frailty population (median SPPB = 5; median age = 86). Some participants may have already been near their functional capacity due to frailty or comorbidities, thus limiting potential gains. While Mobility-Fit did not significantly reduce frailty scores, the stabilisation of frailty status suggests that the intervention may help delay further deterioration. Maintaining baseline function in highly vulnerable populations is itself a clinically meaningful outcome [38]. The modest SPPB change (0.2 points) in the intervention group, though below the threshold for clinically meaningful gains (0.4–1.5 points) established in less impaired cohorts [39], may reflect the challenge of achieving substantial functional improvements in highly frail individuals [40].

The reduction in self-reported fall risk (LASA score) across both groups may reflect improved fall efficacy through structured PA. Enhanced self-efficacy, often mediated by repeated exposure to balance and strength exercises, is a known mediator of fall risk reduction in older adults [41]. A notable finding was the significant improvement in health-related QoL in the Mobility-Fit group, particularly among frail participants. This suggests that the program, combined with its social interaction and cognitive stimulation, substantially impacted perceived well-being [42]. Notably, frail participants in the Mobility-Fit group reported improvements in health-related QoL despite minimal physical gains, underscoring the importance of psychosocial factors in LTC interventions.

The Mobility-Fit program demonstrated high adherence, exceeding the mean rates reported in prior trials (70%) [43]. The difference in adherence rates between the intervention (92%) and control (69%) groups may have contributed to the observed outcomes and should be considered when interpreting the results. This robust engagement likely reflects a confluence of participant-, provider- and system-level facilitators. The program’s tailored design, which adapted exercises to participants’ baseline abilities, may have mitigated barriers related to medical comorbidities and low self-efficacy [44] by minimising perceived exertion. Organisational support was also pivotal: facility leadership ensured scheduling consistency, and PTs received targeted training to deliver progressive, cognitively accessible exercises. Such provider-level engagement aligns with evidence that staff competency and institutional buy-in are key determinants of adherence in LTC settings [45]. The absence of adverse events underscores the program’s safety and feasibility, even for frail participants.

Several limitations warrant caution. First, the specification of multiple primary outcomes increases the risk of Type I error. Second, the 12-week intervention precludes conclusions about long-term effects, as meta-analyses suggest that programs lasting 6 months or longer are more effective in reducing fall rates in this population [46]. Third, baseline imbalances in SPPB scores, though adjusted for statistically, may have confounded mobility outcomes. Fourth, the reliance on self-reported fall risk and QoL may introduce potential response bias. Furthermore, improvements observed at follow-up could be partially attributable to greater familiarity with the assessment procedures or increased participant motivation, factors common in exercise trials. Finally, while subgroup analyses provided valuable insights, the smaller sample size within each frailty category limited the statistical power to detect differential effects.

## Conclusion

The Mobility-Fit program significantly improved upper-limb strength and QoL among older adults in LTC facilities. The similar improvements in lower-limb strength and mobility observed in both groups underscore the fundamental value of structured PA. For clinicians, these findings suggest that incorporating upper-body and core exercises into routine programming can yield meaningful gains in both physical capacity and perceived well-being, supporting a more holistic approach to exercise in LTC settings.

## Supplementary Material

aa-25-1517-File002_afaf349

aa-25-1517-File003_afaf349

aa-25-1517-File004_afaf349

## Data Availability

Please contact the corresponding author for data requests.
