# Supplementary material for: Effects of Mobility-Fit, a tailored multicomponent physical activity program with upper-limb emphasis, on strength, mobility and fall risk among older adults in long-term care: a cluster randomised controlled trial
Source: Age Ageing. 2025 Dec 15;54(12):afaf349. doi: 10.1093/ageing/afaf349 (PMC12704421; doi:10.1093/ageing/afaf349)
Supplement: aa-25-1517-File002_afaf349 [file aa-25-1517-file002_afaf349.docx]

**Title: Effects of Mobility-Fit, a Tailored Multicomponent Physical Activity Program with Upper-Limb Emphasis, on Strength, Mobility, and Fall Risk among Older Adults in Long-Term Care: A Cluster Randomized Controlled Trial**

**Mobility-Fit Program: Weekly Progression with Time Allocation**

| **Week** | **Primary Focus & Milestones** | **Session Structure & Time Allocation (Total ~45 mins)** | **Progression Method** |
| --- | --- | --- | --- |
| **Week 1** (Introduction & Foundation) | **Focus:** Socialization, safety, introducing movement patterns, building confidence. **Milestone:** Participants understand program goals; perform joint mobility in all planes. | • **Welcome (2–5 min)** • **Agility (7–12 min total):** Ball toss/bounce. • **Warm-up (10 min total):** Seated joint mobilization (5 min) → increased ROM & coordination (5 min). • **Posture/Core (3–5 min):** Lumbar extension, seated balance, hand prints. • **Endurance (5 min):** Multiple repetitions of warm-up movements. • **Strength (5–10 min):** Foot prints, shoulder external rotation, heel squeeze. • **Stretch (2 min):** Short holds, full ROM. | • Increase ROM within warm-ups. • Increase repetitions (e.g., 2–4 to 4–6). • Add cognitive challenge to agility. |
| **Week 2** (Building Engagement & Core) | **Focus:** Increased core engagement, introduction of seated balance, longer standing intervals. **Milestone:** Participants sit away from chair back frequently; sustained posture is improved. | • **Welcome (2 min)** • **Agility (6 min total):** Ball toss/bounce progressed to “opposite return”. • **Warm-up (10 min total):** Seated joint mobilization (5 min) → increased ROM & complexity with unsupported sitting (5 min). • **Posture/Core (5 min):** Functional reach, lumbar extension, hand prints.  • **Balance standing (2 min):** Weight shift, static balance, dynamic balance, gait training. • **Endurance (5 min):** Higher repetition movements (4–6 repetitions). • **Functional strength (5–10 min):** Hip extension, slow sitting/foot prints, lat pull, heel/toe raise, shoulder external rotation, pelvic floor, grip. • **Stretch (2–3 min):** Increase repetitions; introduce more stretches. | • Increase duration of unsupported sitting. • Introduce “perceived resistance.” • Add duration and repetitions to strength exercises. |
| **Week 3** (Increasing Standing Time) | **Focus:** Integrating standing as a core component, introducing gait training, and dynamic balance. **Milestone:** Significant portion of the session is performed standing with support. | • **Welcome (2 min)** • **Agility (7-12 min total):** Introduce numbered tasks (1..2..3..). • **Warm-up/Endurance (10–15 min total):** Seated mobilization (5 min) → standing warm-up with arm/leg combinations & quickened pace (5–10 min). • **Posture/Core/Balance (5 min):** Functional reach (standing), weight shift, static standing balance, dynamic balance. • **Strength (5–10 min):** Hip extension, triceps, squats, scapular retraction (standing). • **Stretch (2–3 min):** Increase duration and repetitions. | • Introduce standing for entire components. • Decrease BOS in standing. • Monitor intensity with RPE (4–5). |
| **Week 4** (Integrating Gait & Balance) | **Focus:** Making gait training part of the warm-up, challenging dynamic balance with less support. **Milestone:** Participants perform varied gait patterns and balance challenges with minimal hand support. | • **Welcome (2 min)** • **Agility (4 min total):** Foot placement or numbered tasks. • **Warm-up/Gait/Endurance (15–20 min total):** Seated & standing warm-up integrated with gait training (steps forward, sideways, back, turns). • **Posture/Core (5 min):** Trunk leans, lumbar extension. • **Balance/Gait (7 min total):** Weight shift → static/dynamic balance → gait training. • **Strength (10 min):** Hip extension, slow sitting, lat pull, triceps (with perceived resistance). • **Stretch (2–3 min):** Increase repetitions and duration. | • Integrate gait into warm-up. • Increase stride length/width. • Decrease hand support for balance. |
| **Week 6** (Complexity & Power) | **Focus:** Challenging coordination and memory, introducing eccentric strength and power for fall recovery. **Milestone:** Participants perform multi-tasking movements and practice “brake and recover” maneuvers. | • **Welcome (2 min)** • **Agility (2 min):** Increase speed but ensure good technique. • **Warm-up/Gait/Endurance (15–20 min):** Complex patterns challenging memory and coordination (e.g., marching with directional changes). • **Posture/Core (5 min):** Trunk leans, functional reach. • **Balance/Gait (7 min):** Weight shift to static balance, dynamic balance with marching pauses. • **Strength (10 min):** Higher level exercises (e.g., lunges, unilateral work) with increased repetitions/sets. • **Stretch (2–3 min):** Full routine. | • Increase complexity of coordination (multi-tasking). • Introduce eccentric & power training (“brake and recover”). • Increase load (light resistance). |
| **Week 12** (Final Progression) | **Focus:** Combining all elements into a continuous, standing-dominated session. **Milestone:** Participants complete a session with advanced balance, strength, and agility tasks. | • **Welcome (2 min)** • **Warm-up/Gait/Endurance (10 min):** Continuous movement with gait, turns, directional changes, and steps over blocks. • **Balance (3–5 min):** Multi-directional weight shift, tandem walk, walk on toes/heels. • **Agility (3–5 min):** Participant’s choice, focusing on speed and reaction. • **Posture/Core/Strength (20 min):** Combined block of advanced exercises with applied resistance (e.g., lat pull, lunges, unilateral hip abduction). • **Stretch (3–5 min):** Increased duration and emphasis on final posture. | • Apply resistance as appropriate. • Decrease BOS during strength exercises. • Focus on multi-plane, functional movements. |

*Notes: This intervention detail table provides a generalized overview of the Mobility-Fit program’s progression. It is intended for informational purposes only. For each participant, the program must be individually tailored. A mandatory assessment by the physiotherapists is essential before commencing the intervention. The physiotherapists determined the appropriate starting level, exercises, and rate of progression based on the individual’s specific strengths, limitations, and medical conditions. All exercises and progressions should be adapted accordingly to ensure safety and effectiveness. Abbreviations: BOS = base of support; ROM = range of motion; RPE = rate of perceived exertion.*
