# Supplementary material for: Effects of Mobility-Fit, a tailored multicomponent physical activity program with upper-limb emphasis, on strength, mobility and fall risk among older adults in long-term care: a cluster randomised controlled trial
Source: Age Ageing. 2025 Dec 15;54(12):afaf349. doi: 10.1093/ageing/afaf349 (PMC12704421; doi:10.1093/ageing/afaf349)
Supplement: aa-25-1517-File003_afaf349 [file aa-25-1517-file003_afaf349.docx]

**Title: Effects of Mobility-Fit, a Tailored Multicomponent Physical Activity Program with Upper-Limb Emphasis, on Strength, Mobility, and Fall Risk among Older Adults in Long-Term Care: A Cluster Randomized Controlled Trial**

**Supplementary text:**

**Data Analysis**

Subgroup analyses were performed based on frailty status (Non-frail vs. Frail), using individual participants as the unit of analysis. Interaction terms in the generalized estimating equation models were used to examine whether the intervention effect differed by frailty status. Due to the relatively small sample size in the subgroup, clustering was not considered in this analysis.

**Results**

Subgroup analyses based on frailty status (assessed using the FRAIL-NH scale) revealed differential effects (see Table 1 in Appendix 2). Among non-frail older adults, significant Group×Time interactions were observed for elbow extension strength, the Short Physical Performance Battery (SPPB) gait sub-score, EQ-5D utility score, and EQ-VAS. In this subgroup, the intervention group showed greater improvements in elbow extension strength (*β*=0.81, adjusted *p*=0.022), EQ-5D utility score (*β*=0.09, adjusted *p*=0.019), and EQ-VAS (*β*=9.32, adjusted *p*=0.021) compared to the control group. In contrast, the control group demonstrated a higher increase in the SPPB gait sub-score (*β*=-0.22, adjusted *p*=0.023).

Among frail older adults, significant Group×Time interactions were observed for elbow flexion strength and the EQ-5D utility score. The intervention group exhibited a significant increase in the EQ-5D utility scores (*β*=0.15, adjusted *p*=0.020), whereas the control group showed a greater improvement in elbow flexion strength (*β*=-0.89, adjusted *p*=0.046).

**Discussion**

Subgroup analyses revealed frailty-specific effects. Among non-frail participants, the intervention group exhibited greater improvements in elbow extension strength and health-related quality of life, while the control group showed a larger increase in SPPB gait performance after 12 weeks. This suggests that non-frail residents derived both functional and psychosocial benefits from Mobility-Fit, whereas standard care primarily improved lower-limb mobility.

In frail participants, the intervention group showed improved EQ-5D utility scores, while the control group exhibited higher gains in elbow flexion strength. This contrast aligns with the nonlinear impact of frailty on intervention efficacy [1, 2]. Non-frail residents, possessing greater physiological reserve, may be able to translate multicomponent training into broad functional and psychological gains [1]. For frail individuals, the isolated improvement in elbow flexion strength observed in the control group may be attributable to more severe baseline mobility impairments in the intervention group, which could have limited their engagement in training. Additionally, these gains may reflect compensatory upper-limb use during seated exercises (e.g., grasping armrests for stability) or functional transfers, a phenomenon observed in deconditioned populations where even minimal resistance training can yield measurable improvements [3].

Despite limited physical gains, frail participants in the Mobility-Fit group experienced psychosocial benefits, emphasizing the program’s ability to enhance well-being. Given their multimorbidity and reduced physiological reserve, frail residents may require adjunctive strategies (e.g., nutritional support) to achieve physical gains comparable to those of non-frail individuals [4]. These findings advocate for frailty-adapted interventions that balance functional and psychosocial well-being in long-term care settings.

**Table 1. Subgroup comparisons (Non-frail vs. Frail) for effects of the Mobility-Fit training on primary and secondary outcomes by adjusted Generalized Estimating Equation model.**

| **Variable** | **Non-frail (n = 76)** | | | | | | | **Frail (n = 70)** | | | | | | |
| --- | --- | --- | --- | --- | --- | --- | --- | --- | --- | --- | --- | --- | --- | --- |
|  | **Group × Time interaction** | | **Group** | | **Time** | | **Cohen’s d** | **Group × Time interaction** | | **Group** | | **Time** | | **Cohen’s d** |
|  | ***β* (SE)** | ***p*** | ***β* (SE)** | ***p*** | ***β* (SE)** | ***p*** |  | ***β* (SE)** | ***p*** | ***β* (SE)** | ***p*** | ***β* (SE)** | ***p*** |  |
| Elbow flexion strength (kg) | -0.02 (0.54) | 0.976 | 0.16 (0.21) | 0.468 | 0.29 (0.39) | 0.464 | 0.08 | -0.95 (0.45) | **0.038** | 0.06 (0.18) | 0.749 | 0.29 (0.29) | 0.319 | 0.64 |
| Elbow extension strength (kg) | 0.80 (0.37) | **0.030** | 0.01 (0.12) | 0.959 | 0.13 (0.23) | 0.563 | 0.71 | 0.25 (0.43) | 0.560 | -0.34 (0.17) | **0.040** | 0.83 (0.28) | **0.003** | 0.07 |
| Knee extension strength (kg) | -1.67 (1.04) | 0.107 | 0.38 (0.38) | 0.324 | 3.67 (0.71) | **< 0.001** | 0.41 | 0.34 (1.06) | 0.747 | -0.79 (0.48) | 0.103 | 1.67 (0.81) | **0.040** | 0.14 |
| Handgrip strength-R (kg) | -0.98 (0.57) | 0.086 | 0.31 (0.19) | 0.107 | 0.58 (0.41) | 0.152 | 0.39 | 1.02 (0.72) | 0.159 | -0.58 (0.29) | **0.047** | -0.11 (0.52) | 0.828 | 0.21 |
| Handgrip strength-L (kg) | -0.50 (0.54) | 0.363 | 0.28 (0.16) | 0.077 | -0.01 (0.40) | 0.986 | 0.13 | 0.89 (0.68) | 0.188 | -0.14 (0.24) | 0.553 | -0.11 (0.46) | 0.814 | 0.38 |
| Reaction time (s) | 0.01 (0.07) | 0.839 | -0.01 (0.02) | 0.836 | -0.04 (0.04) | 0.343 | 0.05 | 0.03 (0.09) | 0.702 | -0.01 (0.05) | 0.843 | -0.12 (0.06) | **0.040** | 0.09 |
| Postural sway-AP (mm) | -1.21 (1.52) | 0.425 | -0.39 (0.46) | 0.395 | 2.07 (1.13) | 0.067 | 0.34 | -3.59 (2.60) | 0.167 | -0.36 (1.09) | 0.740 | 2.64 (1.76) | 0.133 | 0.51 |
| Postural sway-ML (mm) | -6.98 (3.56) | 0.050 | 0.73 (1.50) | 0.627 | 1.06 (2.74) | 0.698 | 0.57 | -0.79 (5.49) | 0.886 | -0.33 (2.74) | 0.905 | -2.00 (3.92) | 0.610 | 0.07 |
| STS time (s) | 0.91 (1.41) | 0.520 | 0.03 (0.72) | 0.968 | -4.11 (1.04) | **< 0.001** | 0.21 | -1.64 (2.11) | 0.438 | 0.67 (1.02) | 0.514 | -3.35 (1.54) | **0.030** | 0.16 |
| Walking speed (m/s) | -0.02 (0.03) | 0.423 | -0.01 (0.01) | 0.892 | -0.01 (0.02) | 0.517 | 0.26 | 0.01 (0.04) | 0.808 | -0.01 (0.01) | 0.384 | 0.03 (0.02) | 0.192 | 0.02 |
| SPPB-Total (point) | -0.42 (0.32) | 0.193 | 0.16 (0.11) | 0.136 | 0.74 (0.24) | **0.002** | 0.26 | -0.39 (0.36) | 0.283 | 0.10 (0.15) | 0.485 | 0.44 (0.23) | **0.048** | 0.27 |
| SPPB-STS (point) | -0.13 (0.19) | 0.517 | 0.03 (0.05) | 0.530 | 0.43 (0.14) | **0.002** | 0.16 | 0.05 (0.16) | 0.770 | -0.04 (0.05) | 0.418 | 0.08 (0.11) | 0.460 | 0.01 |
| SPPB-Gait (point) | -0.25 (0.10) | **0.014** | 0.03 (0.03) | 0.268 | 0.07 (0.05) | 0.142 | 0.67 | 0.01 (0.12) | 0.966 | -0.02 (0.05) | 0.646 | 0.08 (0.07) | 0.290 | 0.05 |
| SPPB-Balance (point) | -0.07 (0.21) | 0.722 | 0.14 (0.09) | 0.134 | 0.23 (0.16) | 0.161 | 0.10 | -0.43 (0.26) | 0.094 | 0.21 (0.13) | 0.119 | 0.28 (0.17) | 0.105 | 0.29 |
| LASA (point) | 0.65 (0.55) | 0.238 | 0.01 (0.22) | 0.953 | -2.10 (0.40) | **< 0.001** | 0.39 | -0.52 (0.70) | 0.462 | 0.35 (0.27) | 0.204 | -0.26 (0.47) | 0.583 | 0.08 |
| EQ-5D utility score | 0.12 (0.04) | **0.005** | -0.04 (0.02) | 0.084 | -0.03 (0.03) | 0.321 | 0.65 | 0.22 (0.06) | **< 0.001** | -0.07 (0.04) | 0.067 | -0.01 (0.04) | 0.818 | 0.77 |
| EQ-VAS | 10.40 (4.36) | **0.017** | -1.08 (1.90) | 0.569 | -4.35 (3.17) | 0.168 | 0.69 | 5.30 (4.79) | 0.268 | -1.75 (2.12) | 0.409 | -1.43 (3.18) | 0.653 | 0.25 |

*Notes: Adjusted by age, sex, body mass index, walking aid used, and baseline value. Bold p value represents being statistically significant.*

*Abbreviations: AP = Antero-posterior; EQ-5D = EuroQol five-dimension questionnaire; EQ-VAS = EuroQol Visual Analogue Scale; FRAIL-NH = 7-item FRAIL-Nursing Home Scale; L = Left; LASA = Longitudinal Aging Study Amsterdam fall risk profile questionnaire; ML = Medio-lateral; R = Right; SE = Standard error; SPPB = Short Physical Performance Battery; STS = Sit-To-Stand.*

**References:**

1. Fried LP, Tangen CM, Walston J, et al. Frailty in older adults: evidence for a phenotype. *J Gerontol A Biol Sci Med Sci* 2001; 56: M146-M57.

2. Dent E, Martin FC, Bergman H, et al. Management of frailty: opportunities, challenges, and future directions. *Lancet* 2019; 394: 1376-86.

3. Kato Y, Islam MM, Koizumi D, et al. Effects of a 12-week marching in place and chair rise daily exercise intervention on ADL and functional mobility in frail older adults. *J Phys Ther Sci* 2018; 30: 549-54.

4. Lorbergs A, Prorok J, Holroyd-Leduc J, et al. Nutrition and physical activity clinical practice guidelines for older adults living with frailty. *J Frality Aging* 2022; 11: 3-11.
