# Supplementary material for: Effects of Mobility-Fit, a tailored multicomponent physical activity program with upper-limb emphasis, on strength, mobility and fall risk among older adults in long-term care: a cluster randomised controlled trial
Source: Age Ageing. 2025 Dec 15;54(12):afaf349. doi: 10.1093/ageing/afaf349 (PMC12704421; doi:10.1093/ageing/afaf349)
Supplement: aa-25-1517-File004_afaf349 [file aa-25-1517-file004_afaf349.docx]

**Title: Effects of Mobility-Fit, a Tailored Multicomponent Physical Activity Program with Upper-Limb Emphasis, on Strength, Mobility, and Fall Risk among Older Adults in Long-Term Care: A Cluster Randomized Controlled Trial**

Table 1. Effects of the Mobility-Fit training on primary and secondary outcomes by adjusted Generalized Estimating Equation model (per-protocol sample, intervention group: n=55; control group: n=56).

| Variables | Group × Time interaction | | Group  (Intervention vs. Control) | | Time  (12 weeks vs. Baseline) | | Cohen’s d |
| --- | --- | --- | --- | --- | --- | --- | --- |
|  | *β* (SE) | *p* | *β* (SE) | *p* | *β* (SE) | *p* |  |
| Elbow flexion strength (kg) | -0.31 (0.44) | 0.478 | 0.05 (0.15) | 0.738 | 0.33 (0.35) | 0.349 | 0.15 |
| Elbow extension strength (kg) | 0.60 (0.32) | 0.064 | -0.13 (0.13) | 0.302 | 0.37 (0.23) | 0.112 | 0.34 |
| Knee extension strength (kg) | -1.23 (1.10) | 0.263 | -0.05 (0.33) | 0.870 | 2.72 (0.92) | **0.003** | 0.37 |
| Handgrip strength-R (kg) | -0.14 (0.70) | 0.839 | 0.07 (0.15) | 0.621 | 0.03 (0.49) | 0.952 | 0.03 |
| Handgrip strength-L (kg) | 0.13 (0.66) | 0.839 | 0.16 (0.16) | 0.291 | -0.09 (0.45) | 0.840 | 0.14 |
| Reaction time (s) | 0.08 (0.06) | 0.199 | -0.03 (0.03) | 0.370 | -0.08 (0.05) | 0.146 | 0.21 |
| Postural sway-AP (mm) | -2.72 (1.94) | 0.160 | -0.05 (0.69) | 0.941 | 3.00 (1.10) | **0.006** | 0.41 |
| Postural sway-ML (mm) | -3.62 (4.07) | 0.374 | 0.23 (2.01) | 0.910 | -0.36 (2.98) | 0.905 | 0.23 |
| STS time (s) | -0.52 (1.58) | 0.742 | 0.12 (0.67) | 0.863 | -3.27 (0.92) | **< 0.001** | 0.07 |
| Walking speed (m/s) | -0.02 (0.04) | 0.677 | 0.01 (0.01) | 0.853 | 0.02 (0.02) | 0.287 | 0.12 |
| SPPB-Total (point) | -0.26 (0.37) | 0.484 | 0.14 (0.08) | 0.062 | 0.51 (0.24) | **0.034** | 0.10 |
| SPPB-STS (point) | 0.03 (0.15) | 0.834 | -0.02 (0.02) | 0.425 | 0.30 (0.09) | **0.001** | 0.02 |
| SPPB-Gait (point) | -0.13 (0.09) | 0.164 | 0.01 (0.03) | 0.692 | 0.08 (0.05) | 0.113 | 0.35 |
| SPPB-Balance (point) | -0.19 (0.23) | 0.417 | 0.21 (0.09) | **0.014** | 0.14 (0.17) | 0.422 | 0.03 |
| LASA (point) | 0.26 (0.58) | 0.648 | 0.08 (0.20) | 0.704 | -1.34 (0.32) | **< 0.001** | 0.16 |
| EQ-5D utility score | 0.14 (0.05) | **0.001** | -0.04 (0.03) | 0.162 | -0.01 (0.03) | 0.695 | 0.62 |
| EQ-VAS | 7.14 (3.54) | **0.044** | -0.22 (1.48) | 0.884 | -2.41 (2.08) | 0.247 | 0.46 |
| FRAIL-NH (point) | -0.43 (0.42) | 0.314 | 0.02 (0.08) | 0.827 | 0.06 (0.24) | 0.787 | 0.39 |

*Notes: Adjusted by age, sex, body mass index, walking aid used, and baseline values. Bold p-value represents statistical significance.*

*Abbreviations: AP = Antero-posterior; EQ-5D = EuroQol five-dimension questionnaire; EQ-VAS = EuroQol Visual Analogue Scale; FRAIL-NH = 7-item FRAIL-Nursing Home Scale; L = Left; LASA = Longitudinal Aging Study Amsterdam fall risk profile questionnaire; ML = Medio-lateral; R = Right; SE = Standard error; SPPB = Short Physical Performance Battery; STS = Sit-To-Stand.*
